# Sick Leave and Intention to Quit the Job among Nursing Staff in German Hospitals during the COVID-19 Pandemic

**DOI:** 10.3390/ijerph19041947

**Published:** 2022-02-10

**Authors:** Caterina Schug, Franziska Geiser, Nina Hiebel, Petra Beschoner, Lucia Jerg-Bretzke, Christian Albus, Kerstin Weidner, Eva Morawa, Yesim Erim

**Affiliations:** 1 Department of Psychosomatic Medicine and Psychotherapy, University Hospital of Erlangen, Friedrich-Alexander University Erlangen-Nürnberg (FAU), 91054 Erlangen, Germany; eva.morawa@uk-erlangen.de (E.M.); yesim.erim@uk-erlangen.de (Y.E.); 2 Department of Psychosomatic Medicine and Psychotherapy, University Clinic of Bonn, 53127 Bonn, Germany; franziska.geiser@ukbonn.de (F.G.); ninahiebel@web.de (N.H.); 3 Department of Psychosomatic Medicine and Psychotherapy, Ulm University Medical Center, 89081 Ulm, Germany; petra.beschoner@uniklinik-ulm.de (P.B.); lucia.bretzke@uni-ulm.de (L.J.-B.); 4 Department of Psychosomatics and Psychotherapy, Medical Faculty and University Hospital, University of Cologne, 50931 Cologne, Germany; christian.albus@uk-koeln.de; 5 Department of Psychotherapy and Psychosomatic Medicine, Faculty of Medicine, Technische Universität Dresden, 01307 Dresden, Germany; kerstin.weidner@uniklinikum-dresden.de

**Keywords:** nurses, COVID-19, health care, health care workers, sick leave, turnover, intention to quit

## Abstract

Background: Sick leave and turnover of nurses exacerbate an already existing nursing shortage during the COVID-19 pandemic in Germany and other countries. Frequency and associated factors of sick leave and intention to quit among nurses need to be examined to maintain healthcare. Methods: An online survey among nursing staff (N = 757) in German hospitals was conducted between May and July 2021. Sick leave days, intention to quit, working conditions, depression, anxiety and sleep disorder symptoms, effort-reward imbalance (ERI), COVID-19-related and sociodemographic variables were measured. Regression analyses were performed. Results: The intention to quit was present in 18.9%. One third (32.5%) reported sick leave of ≥10 and 12.3% more than 25 days in 12 months. Significant predictors for ≥10 sick leave days were infection with SARS-CoV-2, a pre-existing illness, exhaustion, trust in colleagues and fear of becoming infected. Higher ERI reward levels, perception of sufficient staff and contact with infected patients were associated with lower odds for ≥10 sick leave days. Lower reward levels, having changed work departments during the pandemic, working part-time and higher depression levels significantly predicted turnover intention. Conclusion: Alarmingly, many nurses intend to quit working in healthcare. Perceived reward seems to buffer both sick leave and turnover intention. Enhancing protection from COVID-19 and reducing workload might also prevent sick leave. Depression prevention, improved change management and support of part-time workers could contribute to reducing turnover intention among nurses.

## 1. Introduction

Many countries in the world face a shortage of healthcare workers. In Germany, around 1.7 million professionals work in the field of nursing and caregiving ([1], numbers as of 2019). There are estimations that this is at least 35,000 persons too low to provide nursing care for all in need [2] and, in 2035, there will be an estimated lack of 500,000 professionals in the nursing field in Germany [3].

Working in the field of nursing is generally demanding on a physical (e.g., [4,5]) and psychological level (e.g., [6,7]). The job demands are additionally aggravated by the COVID-19 pandemic increasing the number of patients [8], workload, uncertainty, the risk of infection and the confrontation with suffering and death at the workplace [6]. The COVID-19 pandemic also leads to an elevated psychological burden for those working in the healthcare sector [9] with nursing staff showing higher prevalence of depression and anxiety than other frontline healthcare workers [10].

The nursing profession is associated with a higher number of sick leave days compared to other professions in the healthcare sector [11] and to other professions in general [12]. The total number of sick leave days and the difference between nursing staff and other professions increased due to the COVID-19 pandemic (a mean of 7.0% of all work days were sick leave days among nursing staff vs. 5.4% in other professions [12]). In 2020, employees in nursing in Germany recorded an average of 22.4 days of absence from work due to sickness, while employees in other fields recorded nearly 8 days less (14,6; [13]; long sickness absences might distort the means). Nursing staff is on sick leave more often and longer compared to other occupational groups [13]. The incapacity to work can result from physical as well as from mental health problems. The main causes of sick leave among employees in Germany in 2020 were musculoskeletal problems (22.1% of absences) and mental health problems (17.6%) [14]. Nursing professionals are even more likely to be on sick leave due to musculoskeletal and mental health causes [13] and due to COVID-19 infection [12] than other professions. In Germany, every employee has the right of paid sick leave days [15].

Beside sick leave, turnover intention is another factor leading to a lack of manpower in the field of nursing. Turnover intention can include the intention to quit the activities at the current organization as well as the intention to leave the professional field. Every nurse turnover causes substantial costs to the healthcare system [16], and both sickness absence and turnover were found to be significantly related to elevated burnout levels among the remaining nurses [17].

### 1.1. Predictors and Correlates for Sick Leave and Turnover among Nurses

According to the systematic review of Daouk-Öyry et al. [18], absences and turnover of nurses are influenced by the following factors. On the job level, high workload [19,20,21,22], high job demands [23,24,25], adverse working conditions, exhausting physical activity [23] and work-family conflict [22,23,25] were found to be associated with higher rates of both absences and turnover among nurses. On the organization level, a higher effort-reward ratio, displaying that efforts exceed rewards, was found to lead to higher rates of absences [26] and turnover [22,25]. Effort-reward imbalance was also found to be negatively correlated with self-rated health [27].

On the individual level, poor physical health [23,28] and elevated burnout levels [17,25,29] were related to turnover and absence among nurses.

On the interpersonal level, various variables regarding the quality of relationships and support felt at the workplace influence sick leave and turnover intention [18].

Josephson et al. [23] found work in geriatric care, experiencing social exclusion by superiors and colleagues, negative perceptions of organizational changes and poor self-rated health to be factors increasing the likelihood of both sickness leave and turnover intention.

A meta-analysis [30] revealed the following predictors for increased likelihood of sick leave among nurses: working as a nursing assistant, working night shifts, being employed in pediatrics or psychiatric units and experiencing poor mental health and fatigue. Job demand also increased the likelihood of sickness absences. Support in the workplace diminished the likelihood of sick leave.

Predictors for an increased likelihood of leaving the nursing profession were being young, being male, having worked less time as a nurse, working as an assistant nurse, having musculoskeletal problems and physical working conditions such as having to transfer heavy patients and having limited access to devices for lifting patients [31].

Turnover intention also seems to have a cultural dimension as workplace support and job satisfaction could reduce turnover intention among Polish nurses, while the work-family conflict influenced turnover intention of nurses in Iran [32].

During the COVID-19 pandemic, an increased level of fear of COVID-19 was associated with increased organizational and professional turnover intention in frontline nurses [33].

### 1.2. Focus of the Present Study

Sick leave as well as turnover intention among nurses threaten the healthcare system in many countries including Germany, which is already lacking professionals in the nursing field while facing the COVID-19 pandemic. Therefore, it is very important to assess sick leave and intention to quit among nursing staff as well as their correlates and predictors in the times of the COVID-19 pandemic. By analyzing data of an online survey among nursing staff in Germany between May and July 2021, the present study aimed to answer the following questions:

1. What is the reported number of sick leave days and the reported intention to leave the job?
2. Which sociodemographic, occupational, COVID-19 related, work related and (mental) health related factors are associated with days of sick leave and intention to leave the job among nurses?

## 2. Materials and Methods

### 2.1. Data Collection

Between 28 May and 16 July 2021, an online survey was distributed. The survey represented the third measuring time of the VOICE study that is part of the egePan Unimed (development, testing and implementation of regionally adaptive care structures and processes for evidence-based pandemic management coordinated by the University Medical Center) project supported by the German Federal Ministry of Education and Research. Therefore, the methods are similar to the first and second survey waves (e.g., [34,35]). The link was spread on social platforms or via email distribution lists for staff of the university hospitals of Erlangen, Bonn, Ulm, Cologne and Dresden. Several municipal hospitals and various professional networks also supported spreading the survey.

The survey had a duration of approximately 15 min and was conducted via Unipark, an academic online survey tool. The survey language was German. Participants created a personal code to identify multiple participations by the same person and to enable data matching for the different assessment waves. Inclusion criteria were a minimum age of 18 years, working in the healthcare sector, working place in Germany and sufficient German language competency. The study was approved by the ethics committee of the University Hospital of Erlangen (reference number: 133_20 B) and the involved hospitals.

After removing 62 cases due to identical codes from the dataset, complete data of 3463 healthcare workers remained. Of those, 757 nursing professionals in German hospitals were analyzed in this study.

### 2.2. Measures

#### 2.2.1. Days of Sick Leave and Turnover Intention

The number of sick leave days was assessed by the item “How many whole days did you stay away from work due to illness in the last 12 months?”. The answer options were “None at all/Maximum 9 days/10–24 days/25–99 days/100–365 days”. We used this item based on the fifth item of the German version of the Work Ability Index [36] published by the German Federal Institute for Occupational Safety and Health [37].

Intention to leave the job was measured by the item “Have you felt so stressed due to job-related factors in the last three months, that you plan to quit your job within the medical field?”. We also asked whether participants had the intention to reduce work hours or change the job within the medical field and whether they had already reduced working hours or changed the job within the medical field in the last three months. The answer options of all five items were “yes” or “no”.

#### 2.2.2. Work-Related Variables

Effort and reward were measured by ten items of the effort-reward imbalance scale (ERI; [38]). The effort scale included three items such as “I have constant time pressure due to a heavy work load”. The ERI reward scale included seven items such as “I receive the respect I deserve from my superior or a respective relevant person”. The reward scale also includes aspects of income and promotion prospects.

The ERI ratio is calculated by the division of the two scores (effort E and reward R) including a correction C to account for the unequal number of items: E/(R*C); C = 3/7 = 0.4286. A ratio >1 means that perceived efforts exceed the perceived rewards.

Trust in colleagues was assessed by the item “I can rely on my colleagues when it gets difficult at work”. The answer could be rated on a scale from “0 = I strongly disagree” to “4 = I strongly agree”.

On the same scale, the items “I work more than before the COVID-19 pandemic” and “There is sufficient staff for the current workload” were rated.

Participants also indicated their level of perceived protection against COVID-19 by measures of their employing hospital from “0 = I strongly disagree” to “4 = I strongly agree”.

#### 2.2.3. COVID-19 Related Variables

Fear of infection was assessed with the self-generated item “I was afraid to become infected” on a Likert scale from 0 = “strongly disagree” to 4 = “strongly agree” with regard to the last two weeks.

Furthermore, the following COVID-19 related variables were measured by one item each: having been infected by SARS-CoV-2 (yes/no/I do not know), change of department during the pandemic (yes/no), having had direct contact at work with COVID-19 infected patients (yes/no), being in a risk group because of a chronic illness and workload of the department (from 1 = “strongly below average” to 5 = “strongly above average”).

#### 2.2.4. Mental Health Variables and Exhaustion

The ultra-short screening instrument for depression PHQ-2 (Patient Health Questionnaire; [39]) was used to measure depression symptoms. Participants were asked how often they experienced “feeling down, depressed, or hopeless” and “little interest or pleasure in doing things” over the last two weeks, and they answered on a Likert scale from 0 = “not at all” to 3 = “nearly every day”. Scores ≥3 are considered likely cases of clinically relevant depression [40]. Cronbach’s Alpha for the PHQ-2 was 0.79.

Anxiety symptoms were assessed with the ultra-short screening for generalized anxiety GAD-2 [39]. Participants answered how often they experienced “feeling nervous, anxious or on edge” and “not being able to stop or control worrying” over the last two weeks on a Likert scale from 0 = “not at all” to 3 = “nearly every day”. Scores ≥3 are considered likely cases of clinically relevant generalized anxiety [41]. Cronbach’s Alpha for GAD-2 was 0.82.

Symptoms of sleeping disorders and those of exhaustion were assessed by the items “I suffered from sleep problems” and “I felt physically or mentally exhausted” with regard to the last two weeks and on a scale from “0 = I strongly disagree” to “4 = I strongly agree”.

#### 2.2.5. Sociodemographic and Occupational Variables

The online survey also assessed sociodemographic data such as gender, age (categories: 18–30/31–40/41–50/51–60/>60 years), living alone (or not), having children (or not) and migration background (present if the participant or at least one parent did not have German citizenship by birth [42]) and caring for old, ill or disabled relatives (yes/no). Occupational characteristics were work setting, years of professional experience, area of activity and working full-time or part-time.

#### 2.2.6. Statistical Analysis

Using SPSS V.28 (IBM Deutschland GmbH, Ehningen, Baden-Württemberg, Germany), absolute and relative frequencies and chi-square tests including the effect size measures, Cramer’s V and Phi (≥0.1 = small, ≥0.3 = medium and ≥0.5 = large effect size; based on [43]) were calculated. Furthermore, two binary logistic regressions were performed to identify statistical predictors of sick leave days and intention to quit. Multicollinearity of factors was checked. Significance decisions were based on the alpha error level of 0.05.

All variables described above were included in the regression analysis except for work setting, as it was the hospital setting for the total sample, and except for area of activity. 

## 3. Results

### 3.1. Sample Characteristics

Table 1 and Table 2 show sociodemographic, occupational and COVID-19 related characteristics of the sample.

Of the 757 professionals in nursing, 76.9% were female. The four age categories, 18–30/31–40/41–50/>50 years, were approximately equally frequent. All of the participating nurses worked in a hospital; 86.1% in university hospitals. A total of 26.4% was working in intensive/emergency care. Only 4.2% had less than 3 years of professional experience. The category of more than six years of experience was the most frequent one. The majority of 58.4% were working full-time.

One tenth (10.4%) indicated having been infected with COVID-19, and 28.7% had contact with infected patients at work. A majority of 60.9% reported that the occupancy of their ward was slightly or strongly above average. Every ninth participant (11.0%) had to work in a different department than before/normally due to the pandemic.

### 3.2. Frequency of Sick Leave and Turnover Intention

**Figure 1:**
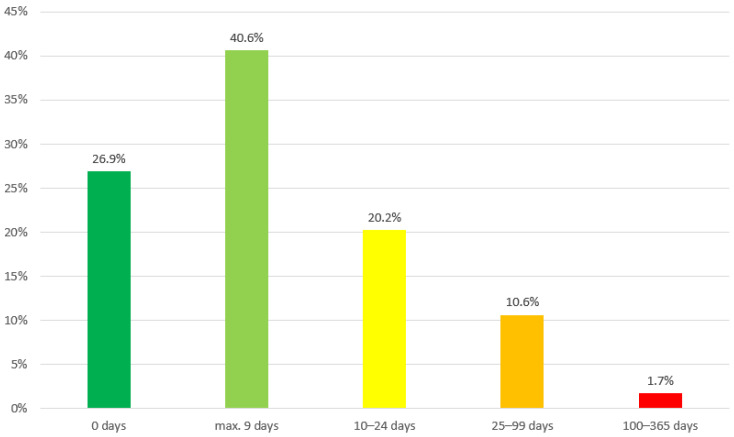
Frequency of sick leave days in the total study sample (N = 757).

As shown in Figure 1, more than one fourth (26.9%) of the sample recorded not one single day of sick leave for the last 12 months. A majority of 40.6% reported a maximum of 9 days of absences due to illness in the last year. This was the most frequent category, and the theoretical median of the sample lies between 1 and 9 days. One fifth (20.2%) reported 10–24 days and 10.6% reported 25–99 days. More than 100 days of absence due to illness were reported by 1.7%. Therefore, one third (32.5%) indicated sick leave of 10 or more days in the last 12 month.

**Figure 2:**
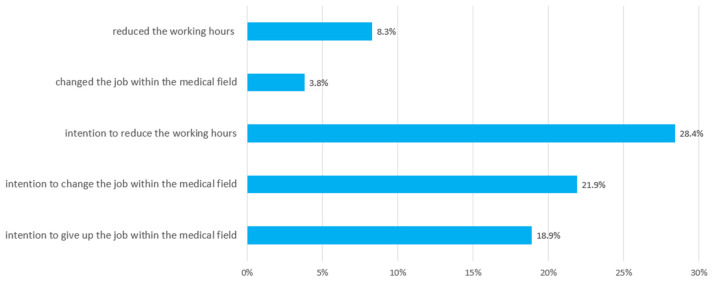
Frequency of nurses’ intention to reduce working hours and to change/give up the job and realized reduction of working hours and change of the job within the medical field (N = 757).

Nearly one fifth (18.9%) of the sample reported the intention to quit working in the medical field (Figure 2). The intention to change jobs within the medical field was present in 21.9%. Over one fourth of the participating nurses (28.4%) planned to reduce their working hours. A total of 8.3% had already reduced their working hours and 3.8% had changed their job within the medical field in the last three months.

### 3.3. Predictors of Sick Leave and Turnover Intention

The binary logistic regression analysis for ≥10 sick leave days as dependent variable revealed an explanation of variance of 20.0% (Table 3). Those who were infected with COVID-19 had almost 7 times elevated odds of having had a sickness absence of 10 days or longer (*p* < 0.001; OR = 6.883 (CI = 3.930–12.055)). Belonging to a COVID-19 risk group due to pre-existing illness (*p* = 0.006; OR = 1.864 (CI = 1.198–2.903)), exhaustion (*p* = 0.012; OR = 1.313 (CI = 1.061–1.624)) and trust in colleagues (*p* = 0.048; OR = 1.205 (CI = 1.002–1.451)) were also significant statistical predictors for an elevated likelihood of ≥10 sick leave days. Those who reported higher levels of fear of becoming infected with COVID-19 showed an elevated chance of ≥10 sick leave days. Their statistical chance of reporting 10 or more days of sick leave was 19.4% higher than for those reporting less fear (*p* = 0.022; OR = 1.194 (CI = 1.026–1.389)).

Lower odds for ≥10 sick leave days were associated with contact with infected patients (*p* = 0.021; OR = 0.635 (CI = 0.432–0.935)). Those who perceived that manpower was sufficient had a chance of 10 or more sick leave days that was reduced by 18% compared to those who did not perceive sufficient staff (*p* = 0.038; OR = 0.818 (CI = 0.676–0.989)). Perceiving higher levels of reward at work reduced the statistical chance of having ≥10 sick leave days by 8.5% (*p* = 0.007; OR = 0.915 (CI = 0.859–0.976)).

The binary logistic regression analysis for the intention to leave the job as dependent variable explained 30.2% of variance (Table 4). The odds of planning to leave the job were more than double in those who had to change their work department compared to those who did not (*p* = 0.003; OR = 2.471 (CI = 1.350–4.523)). Higher levels of depression symptoms (*p* < 0.001; OR = 1.590 (CI = 1.303–1.940)) and working part-time (*p* = 0.009; OR = 1.884 (CI = 1.169–3.037)) were also significant predictors for the intention to leave the job. Reporting higher reward levels diminished the chance of intending to leave the job by 15% (*p* < 0.001; OR = 0.849 (CI = 0.781–0.923)).

The ERI reward scale is the only factor in the models that significantly predicted both sick leave days and the turnover intention.

## 4. Discussion

The aim of the present study was to assess frequency and associated factors of sick leave days and intention to leave the job among nursing staff in Germany during the COVID-19 pandemic.

As two thirds of the sample recorded less than 10 days of sick leave, and 14.6 days of sick leave were found as mean in other professions in 2020 [13], and 22.9 days for nursing staff in 2018 [44], it may be possible that the sample was relatively healthy with regard to sick leave. Nevertheless, more than 12% were on sick leave for more than 25 days in 12 months which is a high jeopardy for the healthcare supply during a pandemic.

Nearly every fifth nursing professional planned to leave the job within the medical field. This is highly alarming considering that there is already a shortage of nursing professionals in Germany [2]. Our findings indicate that this lack seems to expand in the near future entailing adverse consequences for the organization and maintenance of the healthcare system.

For addressing the turnover of nurses as well as long sick leave among them, it is important to know associated factors. As expected, an infection with SARS-CoV-2 and belonging to a COVID-19 risk group due to pre-existing illness were found to be statistical predictors of more days of sick leave. Fear of becoming infected was associated with turnover intention in previous research [33] and with sick leave in this study. The fear of becoming infected could either reflect the actual risk of an infection in the workplace or entail absences as an attempt to protect their own health or both. The fear might also indicate an elevated mental vulnerability or result from more sickness experiences. Less exhaustion and the perception of sufficient staff also expectedly [19,23] predicted less days of sick leave, underlining the importance of healthy workload distribution. The fact that insufficient staff seems to lead to more sick leave days implies a vicious circle of absences. Unexpectedly, trust in colleagues was found to be a predictor of more sickness days. Possibly, trust in colleagues prevents a climate of unhealthy presenteeism [45]. Contact with infected patients also predicted less days of sick leave in the last 12 months. One explanation might be that this group was less prone to developing COVID-19 symptoms in the assessed time period because they might already have had a COVID-19 infection more than one year ago or might be especially cautious regarding infections (possibly including vaccinations) compared to those who do not expect to have contact with infected patients at work. It might also be possible that only especially healthy individuals remained working in the departments with COVID-19 patients and that those more prone to sickness leave had already left these departments. Another imaginable explanation might be that sickness presenteeism is more common in departments providing care for infected patients. Factors associated with presenteeism such as insufficient staff and time pressure [46] () might be present in those departments.

Higher levels of depression were found to be associated with a more likely intention to leave the job which is in line with Daouk-Öyry et al. [18] and with Lo et al. [47] who found that depressive symptoms were linked to the intention to leave the hospital and the profession among nurses in Taiwan. Having changed the ward was also found to be a predictor of the intention to leave the job. This is in line with the finding of Josephson et al. [23] that negative perceptions of organizational changes are related to sick leave and turnover in nurses. Organizational changes in general risk entailing negative affective, cognitive or behavioral reactions such as turnover intention among employees [48]). The review of Oreg et al. [48] highlights the role of organizational trust for successful change processes. Change management including communication and introduction measures in the new team, for example, therefore seem very important regarding turnover intentions. Those who work part-time were also more likely to intend to leave the job. This result had already been found in nursing staff before pandemic times [49]. The compatibility of work and the private/family life might play a role in this relationship as German physicians working in hospitals were less content with the work-family compatibility than those working in other settings, and 86% of them found the possibility to work part-time very important [50]. Given the fact that parents of school children were more burdened than the general public in Germany, at least during the first wave of the COVID-19 pandemic [51], it seems important to support work-family compatibility and provide supportive supervision for those working or aiming to work part-time.

Perceived reward was the only factor found in this study to be (statistically) protective against both sick leave days and intention to leave the job. This is in line with Gräske et al. [52], who reported that nurses with an unfavorable effort-reward imbalance think more often about leaving their occupation, and with Hasselhorn et al. [53], who also reported effort-reward imbalance to be connected to burnout and the intention to leave the nursing profession in nurses in Germany before the pandemic. Altogether, the findings underline the importance of rewards in the nursing profession regarding financial reward, social recognition by leaders, coworkers, patients and society and career opportunities [38]. Future studies should assess which dimensions of reward are the most needed among nurses.

Limitations of the study are that data were self-reported and that the sample was self-selected with the most motivated participating, which is usual in online surveys. Due to the cross-sectional analysis, it is not possible to interpret findings as causalities, nevertheless they deliver indications for future research.

## 5. Conclusions

An alarming proportion of every fifth nursing professional in German hospitals in our study intended to leave the job between May and July 2021. Perceived reward at work seems to buffer both sick leave days and the intention to leave the job. The infection with COVID-19 and the fear of it, as well as insufficient staff and exhaustion, are associated with higher sick leave among nurses. It seems helpful to enhance protection from COVID-19 and a healthy workload distribution. Prevention of depression, sophisticated change management for those who have to change work departments and supervision and support of those aiming to work part-time could be helpful against turnover intention among nursing staff in Germany in pandemic times.

## Figures and Tables

**Table 1:** Sociodemographic and occupational characteristics of the study sample (N = 757) and proportion of sickness leave (≥10 days in last 12 months) and intention to leave the job.

	Total Sample N = 757	Sickness Leave, n (%) (≥10 Days in Last 12 Months)	*p*-Value, Effect Size	Intention to Leave the Job, n (%)	*p*-Value, Effect Size
Gender, n (%)			0.239 * (0.043)		0.247 * (0.042)
Women	582 (76.9)	196 (33.7)		105 (18.0)	
Men	173 (22.9)	50 (28.9)		38 (22.0)	
Diverse	2 (0.3)	0 (0.0)		0 (0.0)	
Age, years, n (%)			0.727 (0.042)		**0.010 (0.123)**
18–30	183 (24.2)	54 (29.5)		43 (23.5)	
31–40	180 (23.8)	58 (32.2)		38 (21.1)	
41–50	158 (20.9)	52 (32.9)		34 (21.5)	
>50	236 (31.2)	82 (34.7)		28 (11.9)	
Living alone, n (%)			0.772 (0.11)		0.377 (0.032)
Yes	195 (25.8)	65 (33.3)		41 (21.0)	
No	562 (74.2)	181 (32.2)		102 (18.1)	
Children, n (%)			0.489 (0.025)		0.388 (0.031)
Yes	374 (49.4)	126 (33.7)		66 (17.6)	
No	383 (50.6)	120 (31.3)		77 (20.1)	
Migration background, n (%)			0.780 (0.010)		0.084 (0.063)
Yes	99 (13.1)	31 (31.3)		25 (25.3)	
No	657 (86.8)	215 (32.7)		118 (18.0)	
Missing	1 (0.1)	-		-	
Caring for old, ill or disabled relatives, n (%)			0.364 (0.033)		0.580 (0.020)
Yes	131 (17.3)	47 (35.9)		27 (20.6)	
No	626 (82.7)	199 (31.8)		116 (18.5)	
Work setting, n (%)			0.518 (0.023)		0.561 (0.021)
University hospital	652 (86.1)	209 (32.1)		121 (18.6)	
Non-university hospital	105 (13.9)	37 (35.2)		22 (21.0)	
Disciplines, n (%)			0.281 (0.091)		0.290 (0.090)
Surgical ward	137 (18.1)	52 (38.0)		32 (23.4)	
Conservative discipline	118 (15.6)	31 (26.3)		17 (14.4)	
Mixed surgical and conservative discipline	93 (12.3)	35 (37.6)		13 (14.0)	
Psychiatry/psychosomatics	100 (13.2)	31 (31.0)		20 (20.0)	
Intensive/emergency care	200 (26.4)	59 (29.5)		43 (21.5)	
Other	109 (14.4)	38 (34.9)		18 (16.5)	
Working in patient care, n (%)			0.632 (0.017)		0.616 (0.018)
Yes	719 (95.0)	235 (32.7)		137 (19.1)	
No	38 (5.0)	11 (28.9)		6 (15.8)	
Professional experience in patient care			0.503 (0.044)		**0.021 (0.104)**
<3 years	32 (4.2)	12 (37.5)		12 (37.5)	
3–6 years	103 (13.6)	29 (28.2)		21 (20.4)	
>6 years	584 (77.1)	194 (33.2)		104 (17.8)	
Missing	38 (5.0)	-		-	
Employment			0.398 (0.031)		0.638 (0.017)
Full-time	442 (58.4)	149 (33.7)		81 (18.3)	
Part-time	315 (41.6)	97 (30.8)		62 (19.7)	

* analyses only refer to male and female; significant *p*-values and corresponding effect sizes are marked in bold.

**Table 2:** COVID-19-related characteristics of the study sample (N = 757).

	Total Sample N = 757	Sickness Leave, n (%) (≥10 Days in Last 12 Months)	*p*-Value, Effect Size	Intention to Leave the Job, n (%)	*p*-Value, Effect Size
Infection with SARS-CoV-2 virus, n (%)			**<0.001 (0.265)**		**0.017 (0.104)**
Yes	79 (10.4)	54 (68.4)		20 (25.3)	
No	600 (79.3)	165 (27.5)		101 (16.8)	
I do not know	78 (10.3)	27 (34.6)		22 (28.2)	
Contact with infected patients, n (%)			0.117 (0.057)		**0.010 (0.093)**
Yes	254 (33.6)	73 (28.7)		61 (24.0)	
No	503 (66.4)	173 (34.4)		82 (16.3)	
Contact with contaminated material, n (%)			0.185 (0.048)		**0.010 (0.094)**
Yes	224 (29.6)	65 (29.0)		55 (24.6)	
No	533 (70.4)	181(34.0)		88 (16.5)	
Risk group due to pre-existing illness, n (%)			**<0.001 (0.123)**		**0.004 (0.104)**
Yes	147 (19.4)	65 (44.2)		40 (27.2)	
No	610 (80.6)	181 (29.7)		103 (16.9)	
Occupancy rate of the wards, n (%)			0.441 (0.070)		0.635 (0.056)
Strongly below average	17 (2.2)	9 (52.9)		4 (23.5)	
Slightly below average	52 (6.9)	18 (34.6)		9 (17.3)	
Average	227 (30.0)	74 (32.6)		36 (15.9)	
Slightly above average	235 (31.0)	76 (32.3)		48 (20.4)	
Strongly above average	226 (29.9)	69 (30.5)		46 (20.4)	
Change of department due to the pandemic, n (%)			0.799 (0.009)		**<0.001 (0.155)**
Yes	83 (11.0)	28 (33.7)		30 (36.1)	
No	674 (89.0)	218 (32.3)		113 (16.8)	
Presently working in home office, n (%)			0.957 (0.002)		0.199 (0.051)
Yes (exclusively/partly)	25 (3.3)	8 (32.0)		2 (8.0)	
No	732 (96.7)	238 (32.5)		141 (19.3)	

Significant *p*-values and corresponding effect sizes are marked in bold.

**Table 3:** Binary logistic regression analysis for ≥10 sick leave days as dependent variable.

Independent Variable Nagelkerkes R^2^ = 20.0%; Hosmer-Lemeshow test: χ^2^ = 5.508; df = 8; *p* = 0.702; 2-Log-Likelihood = 792.672						
	Regression Coefficient	Standard Error	Wald	df	*p*-Value	OR (95% CI: Minimum–Maximum)
*Sociodemographic variables*						
Gender (Ref. = men)						
Women	0.119	0.223	0.284	1	0.594	1.126 (0.727–1.743)
Age (Ref. = 18–30 years)						
31–40	0.374	0.330	1.291	1	0.256	1.454 (0.762–2.774)
41–50	0.459	0.365	1.578	1	0.209	1.582 (0.773–3.237)
>50	0.449	0.350	1.644	1	0.200	1.566 (0.789–3.109)
Living alone (Ref. = No)						
Yes	−0.017	0.223	0.006	1	0.940	0.983 (0.636–1.521)
Children (Ref. = No)						
Yes	0.046	0.219	0.045	1	0.832	1.047 (0.682–1.609)
Migration background (Ref. = No)						
Yes	−0.009	0.265	0.001	1	0.974	0.991 (0.590–1.665)
Caring for old, ill or disabled relatives (Ref. = No)						
Yes	0.071	0.238	0.088	1	0.766	1.073 (0.673–1.710)
*Job—related variables*						
Professional experience in patient care (Ref. = <3 years)						
3–6	−0.396	0.476	0.692	1	0.405	0.673 (0.265–1.710)
>6	−0.322	0.481	0.448	1	0.503	0.725 (0.282–1.861)
Employment (Ref. = Full-time)						
Part-time	−0.345	0.194	3.148	1	0.076	0.709 (0.484–1.037)
*COVID-19—related variables*						
Infection with SARS-CoV-2 (Ref. = No)						
Yes	1.929	0.286	45.520	1	**<0.001**	**6.883 (3.930–12.055)**
I don’t know	0.402	0.279	2.072	1	0.150	1.495 (0.865–2.583)
Contact with infected patients (Ref. = No)						
Yes	−0.453	0.197	5.286	1	**0.021**	**0.635 (0.432–0.935)**
Risk group due to pre-existing illness (Ref. = No)						
Yes	0.623	0.226	7.610	1	**0.006**	**1.864 (1.198–2.903)**
Occupancy rate (Ref. = strongly/slightly below average, average)						
Slightly/strongly above average	−0.271	0.206	1.727	1	0.189	0.763 (0.510–1.142)
Change of the department (Ref. = No)						
Yes	−0.033	0.287	0.013	1	0.909	0.968 (0.551–1.699)
*Symptoms*						
PHQ-2 *	−0.004	0.083	0.002	1	0.962	0.996 (0.846–1.173)
GAD-2 *	0.060	0.075	0.645	1	0.422	1.062 (0.917–1.231)
Sleeping disorders #	−0.103	0.082	1.580	1	0.209	0.902 (0.768–1.059)
Exhaustion #	0.272	0.109	6.273	1	**0.012**	**1.313 (1.061–1.624)**
Fear to become infected #	0.177	0.077	5.245	1	**0.022**	**1.194 (1.026–1.389)**
*Work—related variables*						
ERI effort +	−0.099	0.058	2.903	1	0.088	0.906 (0.809–1.015)
ERI reward +	−0.088	0.033	7.340	1	**0.007**	**0.915 (0.859–0.976)**
Trust in colleagues #	0.187	0.094	3.911	1	**0.048**	**1.205 (1.002–1.451)**
Higher workload #	−0.023	0.076	0.095	1	0.758	0.977 (0.842–1.134)
Sufficient staff #	−0.201	0.097	4.321	1	**0.038**	**0.818 (0.676–0.989)**
Measures of the hospital #	0.030	0.091	0.113	1	0.737	1.031 (0.863–1.232)
Constant	0.356	0.944	0.142	1	0.706	1.428

N = 714. OR = odds ratio; CI = confidence interval; PHQ-2 = Patient Health Questionnaire-2; GAD-2 = Generalized Anxiety Disorder-2; ERI = effort-reward imbalance questionnaire; * Scale: 0 = never, 1 = on single days, 2 = on more than half of the days, 3 = almost every day; # Scale: 0 = strongly disagree, 1 = rather disagree, 2 = neither agree or disagree, 3 = rather agree, 4 = strongly agree; + Scale: 1 = strongly disagree, 2 = disagree, 3 = agree, 4 = strongly agree; significant *p*-values and corresponding ORs/CIs are marked bold.

**Table 4:** Binary logistic regression analysis for the intention to leave the job as dependent variable.

Independent Variable Nagelkerkes R^2^ = 30.2%; Hosmer-Lemeshow test: χ^2^ = 2.698; df = 8; *p* = 0.952; 2-Log-Likelihood = 546.477						
	Regression Coefficient	Standard Error	Wald	df	*p*-Value	OR (95% CI: Minimum–Maximum)
*Sociodemographic variables*						
Gender (Ref. = men)						
Women	−0.275	0.271	1.032	1	0.310	0.759 (0.447–1.291)
Age (Ref. = 18–30 years)						
31−40	0.037	0.389	0.009	1	0.924	1.038 (0.484–2.225)
41–50	−0.234	0.439	0.285	1	0.593	0.791 (0.335–1.869)
>50	−0.668	0.442	2.291	1	0.130	0.513 (0.216–1.218)
Living alone (Ref. = No)						
Yes	−0.127	0.279	0.209	1	0.648	0.880 (0.510–1.520)
Children (Ref. = No)						
Yes	0.204	0.278	0.539	1	0.463	1.226 (0.712–2.113)
Migration background (Ref. = No)						
Yes	0.343	0.311	1.216	1	0.270	1.409 (0.766–2.592)
Caring for old, ill or disabled relatives (Ref. = No)						
Yes	0.157	0.290	0.291	1	0.589	1.170 (0.662–2.066)
*Job—related variables*						
Professional experience in patient care (Ref. = <3 years)						
3−6	−0.583	0.531	1.207	1	0.272	0.558 (0.197–1.580)
>6	−0.500	0.542	0.849	1	0.357	0.607 (0.210–1.757)
Employment (Ref. = Full-time)						
Part-time	0.633	0.244	6.763	1	**0.009**	**1.884 (1.169–3.037)**
*COVID-19—related variables*						
Infection with SARS-CoV-2 (Ref. = No)						
Yes	0.537	0.352	2.331	1	0.127	1.711 (0.859–3.411)
I don’t know	0.348	0.333	1.089	1	0.297	1.416 (0.737–2.719)
Contact with infected patients (Ref. = No)						
Yes	0.090	0.234	0.149	1	0.699	1.094 (0.692–1.730)
Risk group due to pre-existing illness (Ref. = No)						
Yes	0.519	0.276	3.538	1	0.060	1.680 (0.978–2.883)
Occupancy rate (Ref. = strongly/slightly below average, average)						
Slightly/strongly above average	−0.241	0.262	0.844	1	0.358	0.786 (0.470–1.314)
Change of the department (Ref. = No)						
Yes	0.905	0.309	8.595	1	**0.003**	**2.471 (1.350–4.523)**
*Symptoms*						
PHQ-2 *	0.464	0.101	20.898	1	**<0.001**	**1.590 (1.303–1.940)**
GAD-2 *	0.029	0.086	0.118	1	0.732	1.030 (0.870–1.219)
Sleeping disorders #	0.091	0.105	0.742	1	0.389	1.095 (0.891–1.346)
Exhaustion #	−0.233	0.149	2.447	1	0.118	0.792 (0.592–1.061)
Fear to become infected #	0.141	0.093	2.295	1	0.130	1.152 (0.959–1.382)
*Work—related variables*						
ERI effort +	−0.017	0.075	0.053	1	0.819	0.983 (0.848–1.139)
ERI reward +	−0.163	0.043	14.658	1	**<0.001**	**0.849 (0.781–0.923)**
Trust in colleagues #	0.103	0.117	0.789	1	0.374	1.109 (0.883–1.394)
Higher workload #	0.167	0.098	2.905	1	0.088	1.181 (0.975–1.431)
Sufficient staff #	−0.118	0.125	0.886	1	0.347	0.889 (0.696–1.136)
Measures of the hospital #	−0.042	0.112	0.139	1	0.709	0.959 (0.770–1.195)
Constant	0.097	1.167	0.007	1	0.934	1.102

N = 714. OR = odds ratio; CI = confidence interval; PHQ-2 = Patient Health Questionnaire-2; GAD-2 = Generalized Anxiety Disorder-2; ERI = effort-reward imbalance questionnaire; * Scale: 0 = never, 1 = on single days, 2 = on more than half of the days, 3 = almost every day; # Scale: 0 = strongly disagree, 1 = rather disagree, 2 = neither agree or disagree, 3 = rather agree, 4 = strongly agree; + Scale: 1 = strongly disagree, 2 = disagree, 3 = agree, 4 = strongly agree; significant *p*-values and corresponding ORs/CIs are marked bold.

## Data Availability

The data presented in this study are available on request from the corresponding author. The data are not publicly available as the approval of the Ethics Committee does not include this type of data publication.
